# High-Frequency Ultrasound Evaluation of the Nail Unit: Essential Insights for Clinicians

**DOI:** 10.7759/cureus.82834

**Published:** 2025-04-23

**Authors:** Claudia Gonzalez, Lauren Valdivia-Muñoz

**Affiliations:** 1 Radiology, Highly Specialized Ultrasound Center, Bogota, COL; 2 Dermatology, Universidad Libre, Cali, COL

**Keywords:** dermatologic ultrasound, high-frecuency ultrasound, nail disorders, onychology, ultrasound

## Abstract

Introduction: High-frequency ultrasound (HFUS) has become increasingly prevalent in dermatological clinical settings over the past decade. However, understanding its appropriate indications and significant utility in onychology is not fully exploited. Based on a clinical self-assessment, the study aimed to provide fundamental recommendations regarding the applications, indications, and limitations of HFUS in nail disorders.

Methods: A clinical practice assessment survey, comprising 15-item multiple-choice questions focused on nail unit ultrasound, was distributed online to clinicians’ members of the Colombian Network of Research and Treatment for Nail Disorders (RITU), during the first quarter of 2024. Participation was voluntary and anonymous; no financial compensation was offered. Aggregated responses were analyzed confidentially.

Results: Around 44 clinicians (86.4% dermatologists and 13.6% radiologists) participated in the study. While most acknowledged the value of HFUS in evaluating nail disorders, knowledge gaps were evident, particularly concerning optimal settings and proficiency maintenance. Despite the widespread use of HFUS, a gap in expertise was observed concerning its application in specific nail pathologies. Although HFUS was the preferred modality for many nail conditions, some concerns about its reliability suggest the need for further training to ensure confident and effective utilization.

Conclusion: This study revealed significant knowledge gaps among clinicians regarding the appropriate application of dermatologic ultrasound and other imaging modalities in the evaluation of nail disorders. The findings emphasize the value and specific indications for HFUS in assessing nail pathology, highlighting the need for enhanced training and education in this specialized area.

## Introduction

Historically, nail disorders (NDs) have been marginalized within the broader dermatological discourse, often managed by non-specialists or even individuals outside the healthcare profession. This neglect has significant implications, not only compromising individual patient care but also limiting our understanding of nail pathology as a reflection of a health issue. However, in recent years, there has been a paradigm shift. Concerted educational initiatives, including dedicated workshops and mentorship programs, have started to mitigate these deficiencies by enhancing clinician proficiency and awareness in onychology and underscoring the increased recognition of its importance. Greater attention to the nail unit (NU) pathology has, in turn, highlighted the diagnostic complexity these conditions pose. Consequently, there is a growing demand for advanced imaging modalities capable of addressing the clinical and diagnostic needs presented by NDs [1-4]. 

NDs, encompassing conditions such as paronychia, psoriasis, and lichen planus, as well as benign and malignant nail tumors, present complex diagnostic challenges for healthcare professionals. These prevalent conditions often necessitate specialized imaging techniques for accurate diagnosis and management. Traditional diagnostic methods frequently lack the resolution required to visualize the intricate subungual anatomy, potentially leading to diagnostic delays and suboptimal treatment strategies [5,6].

High-frequency ultrasound (HFUS) has emerged as a leading modality for the assessment of NDs in this evolution, transforming how these conditions are diagnosed and managed, allowing for precise diagnoses and timely interventions [7]. The initial exploration of the NU using HFUS can be traced back to 1989, when Jemec et al. utilized a 20 MHz transducer to assess the structure of a post-mortem nail plate [8]. This pioneering work was further developed by Hirai et al., who employed a 30 MHz probe to explore nail matrix and nail plate deformities [9]. Their research highlighted the capability of HFUS to provide unparalleled insights into the anatomy of the NU, delivering clear and detailed images of subungual structures, and paving the way for its broader application in dermatological imaging.

Over subsequent years, HFUS technology has undergone significant advancements, with numerous studies validating its efficacy in diagnosing a wide range of nail pathologies, from inflammatory conditions to complex nail tumors [10,11]. This improved diagnostic accuracy has translated into enhanced clinical management and more favorable therapeutic outcomes. HFUS offers the distinct advantage of providing detailed visualization of the NU non-invasively, avoiding the inherent limitations of traditional methods [12,13].

A comprehensive review of the literature on imaging modalities for nail diseases highlights the particular benefits of HFUS. While techniques such as dermoscopy, magnetic resonance imaging (MRI), optical coherence tomography (OCT), and confocal microscopy offer valuable information, HFUS stands out for its utility in evaluating both benign and malignant tumors, chronic inflammatory conditions such as psoriasis and lichen planus, reactive inflammatory processes (e.g., paronychia), and onychocryptosis among many other NDs [14].

Despite its established effectiveness in supporting dermatologists to deliver targeted treatments and enhance patient outcomes, a significant gap exists between the demonstrated capabilities of HFUS and its routine implementation in clinical practice. Bridging this gap is essential to fully realize the potential of this advanced diagnostic modality and improve patient care through timely and accurate diagnoses. 

This study aims to provide fundamental and practical recommendations for the clinical application of HFUS in diagnosing and managing NDs, advocating for a more informed and proactive approach to nail health in the medical community. It is based on a clinical self-assessment conducted among members of the Colombian Network of Research and Treatment for Nail Disorders (RITU), a leading academic group comprising dermatologists, radiologists, and pathologists dedicated to advancing the understanding and treatment of nail diseases in Colombia. The findings presented here are intended to inform clinicians about the practical applications, key indications, and inherent limitations of HFUS in NDs, thereby promoting its integration into dermatology training programs and routine clinical practice and ultimately enhancing patient outcomes.

## Materials and methods

A descriptive, observational, cross-sectional study was conducted at the Highly Specialized Ultrasound Center, Bogota, to assess the current knowledge and application of HFUS in diagnosing and managing NDs. This assessment was facilitated through a 15-item multiple-choice digital survey developed by the research team after a comprehensive review of the medical literature, identifying key topics essential for a thorough understanding of HFUS (Appendix 1). These topics included technical specifications, clinical applications, reliability of results, appropriate requisition formats, and the use of alternative imaging modalities in the diagnosis of NDs. Each question was meticulously crafted and reviewed for clarity and relevance. A pilot test was subsequently conducted with five radiologists to ensure accuracy and comprehension of the survey questions.

The survey was distributed to 71 clinicians who are members of the RITU during the first quarter of 2024. Prior to the distribution of the survey, all participants were informed about the purpose, scope, and nature of the study through a digital informational sheet that accompanied the survey invitation. Clinicians were explicitly asked to consent to participate by checking a mandatory consent box at the beginning of the survey.

Responses were collected anonymously using Google Forms (Google LLC, Mountain View, California, United States), a secure online platform, to ensure participant confidentiality. The data was grouped prior to analysis in Microsoft Excel (Microsoft Corporation, Redmond, Washington, United States). No financial compensation was offered for participation in the study.

Given the study’s observational and descriptive nature, all variables were analyzed as percentages and are presented in absolute frequencies. This method facilitated a straightforward interpretation of the data, highlighting prevalent trends and insights among the surveyed clinicians regarding nail HFUS.

## Results

A total of 44 clinicians completed the survey, comprising 38 dermatologists (86.4%) and six radiologists (13.6%). This section details the questions posed and the correct answers and compares these with the responses provided by the participants, as outlined in Table 1. To facilitate a clearer analysis, questions were categorized by topic area related to NU ultrasound evaluation.

**Table 1:** Ultrasound of the nail unit survey, completed by 44 clinicians * correct response; AP: anteroposterior

Technical Considerations		
Question	Choices	Survey Results (%)
1. What is the imaging method of choice to assess nail diseases?	a. Magnetic resonance imaging (MRI)	a. 0
	b. High frequency ultrasound (HFUS) *	b. 100
	c. X-rays	c. 0
	d. Computed tomography (CT)	d. 0
	e. Diagnostic imaging is not useful in nail diseases.	e. 0
2. What type of transducer should be used to perform nail unit ultrasound?	a. Linear 7 MHz	a. 9.1
	b. Linear 12 MHz	b. 2.3
	c. Convex 12 MHz	c. 0
	d. Linear 15 MHz *	d. 84.1
	e. Linear 70 MHz	e. 4.5
3. High-resolution nail ultrasound should be performed:	a. In grayscale only	a. 2.3
	b. Using Doppler, duplex, color	b. 2.3
	c. In grayscale and using Doppler/duplex color *	c. 95.5
	d. It is not useful to perform nail ultrasound	d. 0
4. For which condition is comparative nail ultrasound always recommended?	a. Retronychia	a. 6.8
	b. Psoriasis	b. 13.6
	c. Lichen planus	c. 2.3
	d. Comparative nail ultrasound should always be performed *	d. 70.5
	e. Comparative nail ultrasound should never be performed	e. 6.8
5. What are the requirements for a proper nail ultrasound assessment?	a. Trained physician in nail disorders	a. 0
	b. High-resolution ultrasound with Doppler analysis	b. 4.5
	c. Copious amount of gel	c. 0
	d. Study in grayscale and color Doppler	d. 0
	e. All the above *	e. 95.5
6. How many assessments per year should a physician complete to demonstrate competence in performing nail ultrasound?	a. 10 studies per year	a. 6.8
	b. 30 studies per year	b. 4
	c. 100 studies per year	c. 50
	d. 300 studies per year *	d. 31.8
Main applications		
7. In which of the following disorders is nail ultrasound indicated?	a. Psoriasis	a. 6.8
	b. Lichen planus	b. 0
	c. Viral wart	c. 0
	d. All of the above *	d. 93.2
	e. None of the above	e. 0
8. In which of the following pathologies is nail ultrasound useful?	a. Benign tumors	a. 0
	b. Malignant tumors	b. 0
	c. Subungual exostoses	c. 0
	d. Myxoid cysts	d. 0
	e. All of the above *	e. 100
	f. None of the above	f. 0
9. In which of the following disorders is nail ultrasound of utility?	a. Retronychia	a. 22.7
	b. Onychomadesis	b. 0
	c. Onychocryptosis	c. 0
	d. All of the above *	d. 72.7
	e. None of the above	e. 4.5
Other radiological imaging for nail disorders		
10. How should an X-ray be requested to study exostoses?	a. Comparative X-ray of the feet	a. 2.3
	b. X-ray of the affected nail unit, AP, and lateral views	b. 9.1
	c. X-ray of the affected nail unit, AP, lateral, and oblique views	c. 27.3
	d. Magnified X-ray of the affected nail unit, AP, lateral, and magnified oblique views *	d. 61.4
11. How should an MRI be requested to study nail diseases?	a. Simple comparative MRI of the feet	a. 0
	b. Contrast-enhanced comparative MRI of the feet	b. 6.8
	c. Non-contrast-enhanced MRI with dedicated foot coil of the affected nail	c. 25
	d. Contrast-enhanced MRI with dedicated foot coil of the affected nail*	d. 68.2
12. In which nail disorders is MRI of the nail unit indicated?	a. Suspected medullary involvement by cancer	a. 2.3
	b. Invasive melanoma	b. 2.3
	c. Osteomyelitis	c. 15.9
	d. All of the above *	d. 77.3
	e. None of the above	e. 2.3
Confidence in the ultrasound of the nail unit		
13. How often do you request a nail unit ultrasound?	a. Frequently	a. 11.6
	b. Rarely	b. 7
	c. Whenever there is an indication	c. 81.4
	d. I do not find ultrasound useful for nail pathology	d. 0
14. How much do you trust the results provided by a high-resolution ultrasound to make decisions for your patient?	a. Always trust	a. 93
	b. Never trust	b. 7

The survey included a final question, number 15, which focused on the demographics of the respondents. Participants were requested to specify their medical specialty. This data helped contextualize the survey responses within the professional backgrounds of the participants, offering further insight into the specialized knowledge and application of HFUS in their respective fields.

HFUS emerged as the unanimous choice, with 100% of the respondents favoring it over other modalities such as MRI, X-ray, and CT, which all garnered 0% preference. This indicates a strong consensus about the superior diagnostic value of HFUS in NDs. The choice of transducer is critical in optimizing ultrasound performance. The linear 15 MHz probe was preferred by 84.1% of participants, reflecting its optimal balance between penetration depth and image resolution suitable for nail assessment. A significant majority (95.5%) indicated that nail HFUS should be performed using grayscale and Doppler/duplex color modes; this dual approach is crucial for detailed visualization of both structural and vascular components of the NU. The survey demonstrated a broad acceptance of HFUS across various NDs, with 93.2% support for its use in conditions like psoriasis, lichen planus, and viral warts. This wide applicability range underscores HFUS’s versatility in dermatology. All participants (100%) affirmed the utility of HFUS in diagnosing pathologies such as subungual exostoses and benign and malignant tumors. This unanimous agreement highlights HFUS’s critical role in the early detection and management of these conditions.

When considering the best radiographic techniques for studying exostoses, 61.4% preferred magnified views, which are essential for detailed bone structure analysis. In MRI applications, 68.2% favored the use of contrast-enhanced scans with a dedicated foot coil, pointing to the necessity for high-resolution images to assess the extent of malignancies and infections effectively.

The confidence in HFUS was overwhelmingly positive, with 93% of the clinicians trusting HFUS to guide clinical decision-making. This high level of trust is indicative of the established reliability and effectiveness of HFUS in clinical practice; however, a small minority (7%) expressed distrust, suggesting areas for improvement in training or technology application.

## Discussion

HFUS has emerged as a pivotal non-invasive tool for evaluating ND, echoing a broad consensus in the literature [5,7,11-15]. In line with earlier reports, the survey results underscore the prevailing role of HFUS over traditional imaging modalities due to its superior anatomical resolution and real-time imaging capabilities. Although conventional radiography and MRI continue to serve as valuable diagnostic resources in particular scenarios, HFUS stands out for its non-invasive nature, lower cost, and ease of use in clinical settings for diagnosing a wide spectrum of nail conditions [16-18]. This observation aligns with previous research, such as the work by Vargas et al., which highlights the increasing reliance on HFUS in clinical settings due to its efficacy and patient-friendly approach [7].

The subsequent sections will explore technical considerations, main applications, additional radiological imaging techniques, and confidence in using ultrasound for the NU. These discussions aim to enhance the optimization of HFUS applications, ensuring maximum diagnostic utility in routine dermatological practice.

Technical considerations

**Figure 1:**
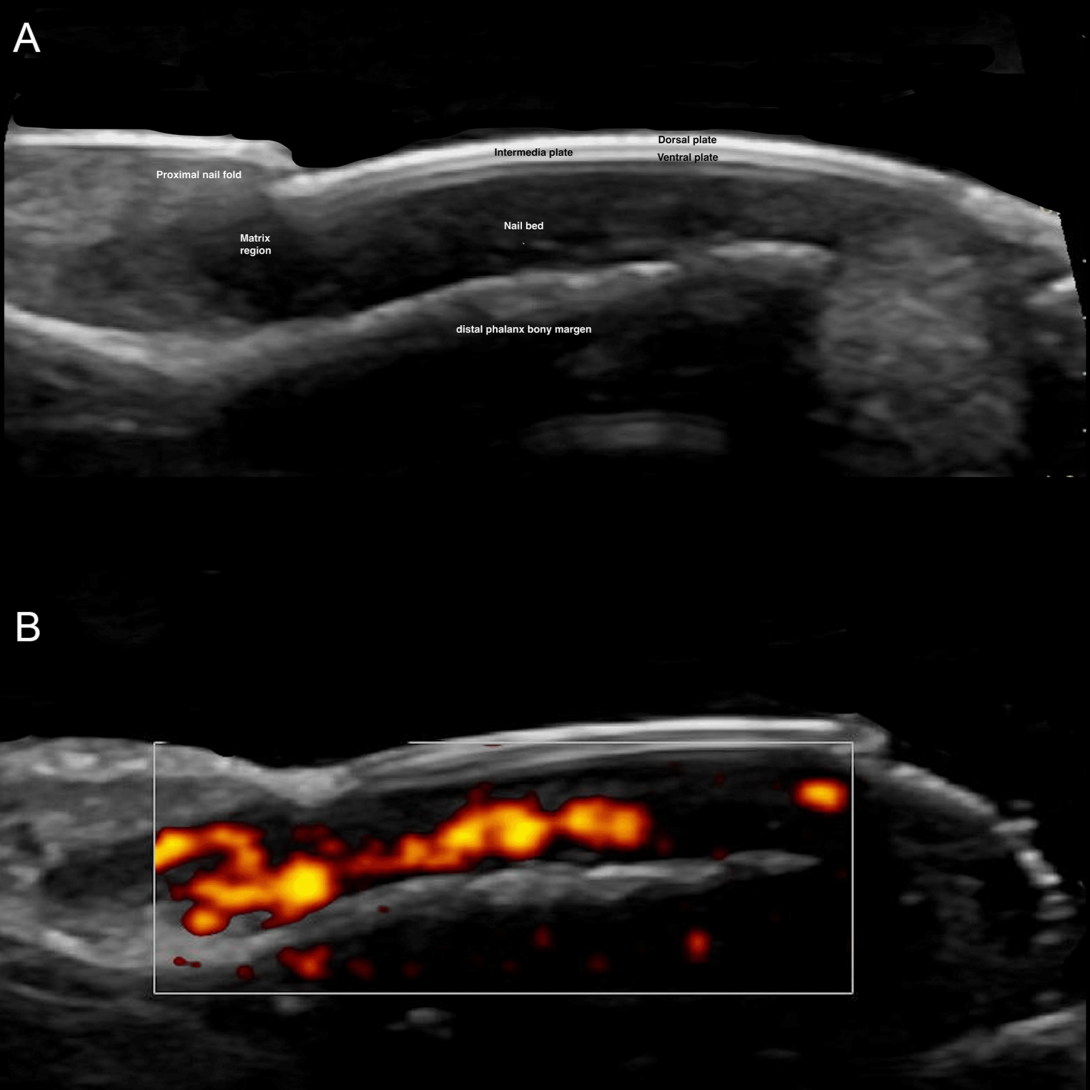
Ultrasound anatomy of the nail unit A: HFUS, a longitudinal greyscale image of the nail unit highlighting all normal anatomical structures with clarity; B: longitudinal Doppler, duplex color ultrasound image of the nail, displaying the normal vascularization. This detailed view highlights the precise flow patterns within the nail unit. Original unpublished image, courtesy of Dr. Claudia Gonzalez. HFUS: high-frequency ultrasound

The unanimous selection of nail unit ultrasound (NU-US) by 100% of the participants as the preferred method for evaluating NDs underscores the significant perceived value clinicians place on the high resolution and dynamic assessment capabilities of HFUS. This preference is not only based on clinical practice but is also well-supported by the existing literature. Studies have consistently demonstrated that HFUS provides superior definition and detailed visualization of NU anatomy, which is crucial for accurate diagnosis and management of NDs. For instance, Jemec et al. (1989), Wortsman et al. (2006), and González CP (2013) have highlighted HFUS's ability to delineate fine anatomical details of the NU that are often missed by conventional imaging techniques, making it indispensable for the assessment of both inflammatory and neoplastic nail conditions [5,8,11]. Additionally, Wortsman et al. (2021), Vargas et al. (2024), and Sechi et al. (2024) reported that HFUS significantly enhances the diagnostic accuracy for subungual tumors and inflammatory diseases by allowing precise localization and characterization of the nail pathology [7,13,14]. These findings are visualized in Figure 1, which demonstrates the superior definition of the NU anatomy provided by high-resolution greyscale ultrasound [14].

Interestingly, while a majority (81%) of participants acknowledged the necessity of using a high-resolution transducer greater than 15 MHz for both greyscale and color Doppler imaging, only 31.8% could correctly identify the recommended number of annual assessments needed to maintain proficiency in nail ultrasound. This discrepancy highlights a significant training gap and suggests a pressing need for structured educational programs to enhance the skills of clinicians using HFUS [6,7,10]. However, it is important to clarify that the requirement to conduct 300 annual studies to achieve competence in HFUS encompasses all aspects of dermatological ultrasound, not limited to the NU alone. 

Moreover, the widespread adoption of comparative nail ultrasound, as supported by 72.7% of participants, is encouraging, as this technique is crucial for accurate diagnosis. This approach of comparing affected nails with healthy controls allows for precise assessment of matrix damage, nail bed alterations, and vascular changes, leading to more accurate diagnoses and tailored treatment plans [5,6,9-14].

Main applications

The high percentage of participants (93.2%) who recognized the value of HFUS in diagnosing inflammatory nail diseases, such as psoriasis, lichen planus, and viral warts, is consistent with existing literature [15-20].

**Figure 2:**
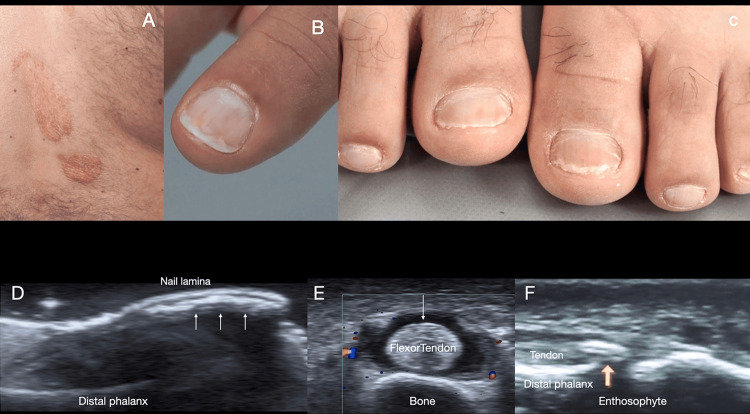
Clinical and ultrasound features of psoriatic onychopathy A: psoriatic skin plaques located in the back region; B,C: nail changes, including oil spots, onycholysis, and an erythematous band proximal to onycholysis evident in both the hands and feet; D: longitudinal grayscale image of the nail unit of the left hallux, nail plate with hyperechoic foci on the ventral plate (white arrows), and increased thickness of the nail bed, characteristics of psoriatic onychopathy; E: axial Doppler, duplex color image of the left thumb, shows laminar collection in the tendon sheath (arrow) indicative of tenosynovitis; F: a longitudinal greyscale image of the right index finger distal interphalangeal joint (yellow arrow) illustrates early changes of psoriatic arthropathy with insertional enthesopathy of the extensor tendon. Original, unpublished image courtesy of Dr. Claudia Gonzalez.

Notably, HFUS can detect subtle intraplate features, such as punctate hyperechoic deposits on the ventral plate, which are present in a majority of patients with psoriatic nail involvement and even in those without obvious nail changes [17-19]. These microstructural changes represent early markers of psoriatic nail disease. Figure 2 showcases clinical images of a 35-year-old male diagnosed with psoriasis, illustrating typical psoriatic skin plaques and nail onychopathy. Further demonstrating the high-resolution ultrasound capabilities, revealing detailed anatomical features of psoriatic onychopathy.

HFUS is also useful for monitoring treatment response in nail psoriasis. Longitudinal studies have found that ultrasound metrics of nail thickness and vascularity diminish with effective therapy. Additionally, HFUS allows for the simultaneous evaluation of enthesitis in structures adjacent to the NU, thereby establishing a connection between nail involvement and the activity of psoriatic arthritis [19-21]. 

Nail lichen planus (NLP) represents another inflammatory disorder where HFUS has proven clinically useful. A recent study identified characteristic features of NLP using HFUS, which include a hypoechoic halo surrounding the proximal nail plate, even in nails appearing clinically unaltered, diffuse thickening and reduced echogenicity of the nail bed, thickening of the proximal nail fold, and significant hypervascularity on Doppler imaging. The study notably concluded that HFUS facilitates the noninvasive diagnosis of NLP, thus eliminating the need for potentially scaring nail biopsies and enabling ongoing monitoring of treatment [22].

An additional principal application of HFUS in onychology is the evaluation of NU tumors. HFUS provides detailed localization, assesses bone involvement, facilitates precise preoperative mapping, and ultimately reduces surgical morbidity [23,24] . In the survey, all respondents acknowledged its utility in identifying a range of suspected neoplastic pathologies within the nail, including glomus tumors, exostoses, mucous cysts, and onychomatricoma. This reflects the significant clinical value of HFUS in this context. Furthermore, the integration of Doppler and duplex color analysis introduces a functional dimension by characterizing the vascular patterns of these tumors, aiding in the differentiation of high-flow vascular tumors from avascular cystic lesions [23-28]. 

In a comprehensive series of 103 cases encompassing common benign and pseudo-malignant nail lesions, such as myxoid cysts, pyogenic granulomas (lobular capillary hemangiomas), fibromas, warts, and glomus tumors, HFUS effectively identified each lesion, revealing distinct morphologic characteristics. HFUS provided new or corrected diagnostic insights in 35% of these cases, substantially influencing the surgical planning process in all cases. Moreover, for certain lesions, HFUS demonstrated greater accuracy than clinical examination alone [23].

**Figure 3:**
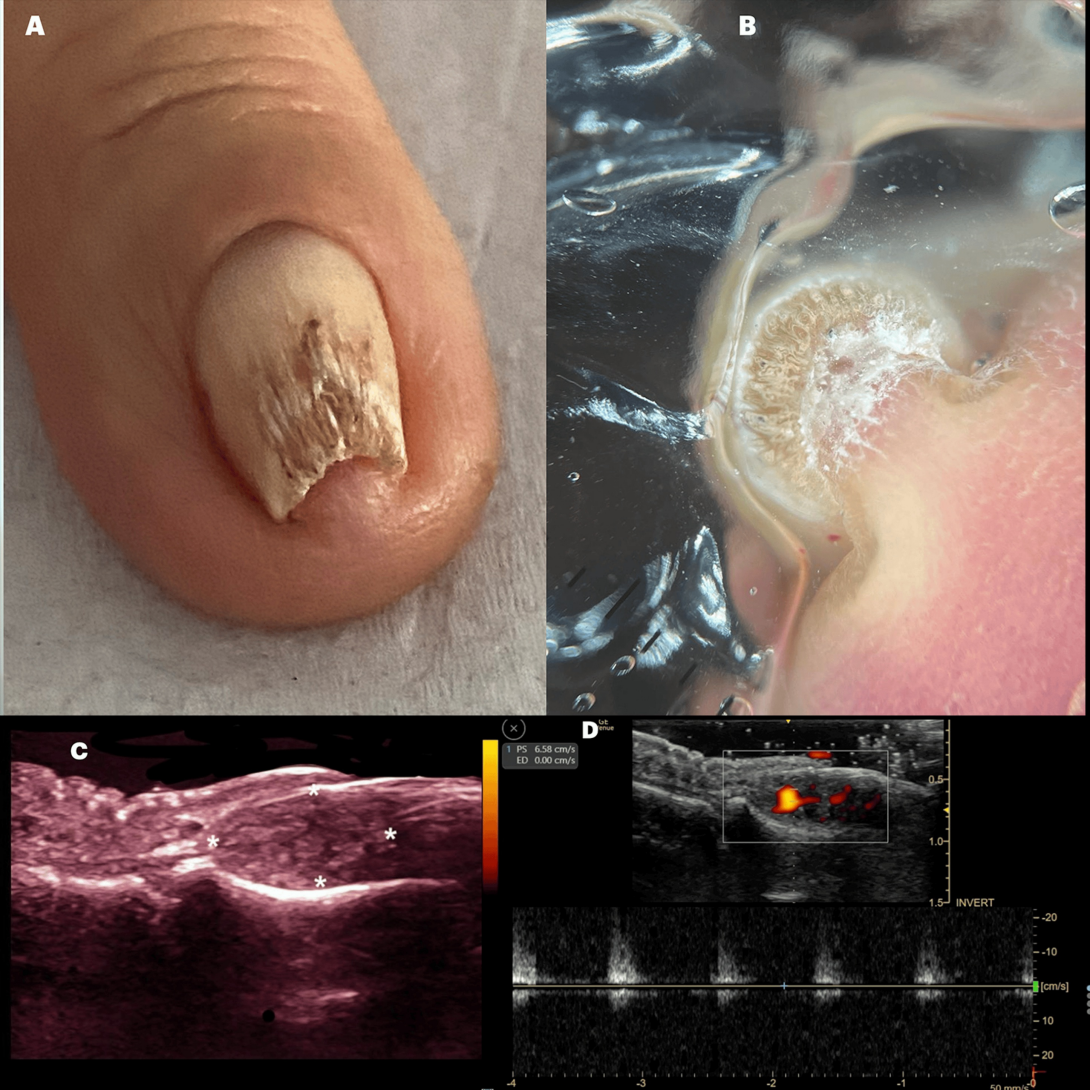
Clinical and ultrasound features of glomus tumor mimicking onychomatricoma A: clinical examination of the index finger reveals total xanthopachyonychia, a pincer nail deformity, and onychodystrophy of the nail plate; B: frontal view dermoscopy of the nail plate reveals a honeycomb pattern suggestive of onychomatricoma (Courtesy of Dr. Isabella Dorado); C: longitudinal greyscale image showing a solid, hyperechoic, expansive mass affecting the matrix area and the proximal third of the nail bed (calipers *), with minimal conditional bone bulges of the distal phalanx; D: Dopler, duplex color analysis highlights the vascularized component of the lesion, diagnosed as a glomus tumor, which was histologically confirmed (Original unpublished image, courtesy of Dr. Claudia Gonzalez).

**Figure 4:**
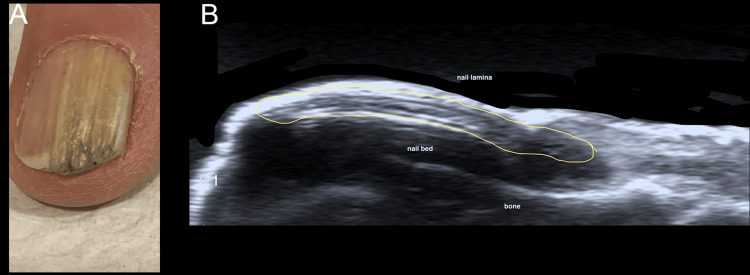
Clinical and ultrasound features of onychomatricoma A: clinical image of the left index showing acquired localized longitudinal xantopachyonychia (band pattern of nail plate thickening with yellow discoloration); B: longitudinal greyscale ultrasound image of the nail delineated by a yellow line showing solid mass, originating above matrix zone and extending along the nail plate with multiple parallel horizontal hyperechogenic lines, characteristic of onychomatricoma. Original unpublished image, courtesy of Dr. Claudia Gonzalez.

**Figure 5:**
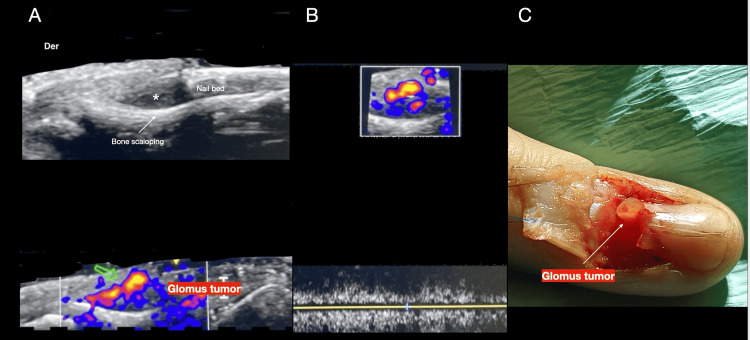
Classic clinical and ultrasound features of glomus tumor A: upper grayscale image displays a hypoechoic nodule in the proximal third of the nail bed and matrix area (asterisk *), and the lower Doppler duplex color image shows an intense vascularized component (green arrow) characteristic of a glomus tumor; B: spectral curve of the lesion illustrates arterial vessels of low resistance (Original unpublished image, courtesy of Dr. Claudia Gonzalez); C: clinical image of the surgical procedure confirming the echographic findings (Courtesy of Dr. Milton Gonzalez).

Figures 3-5 illustrate examples of various nail tumors diagnosed with HFUS.

**Figure 6:**
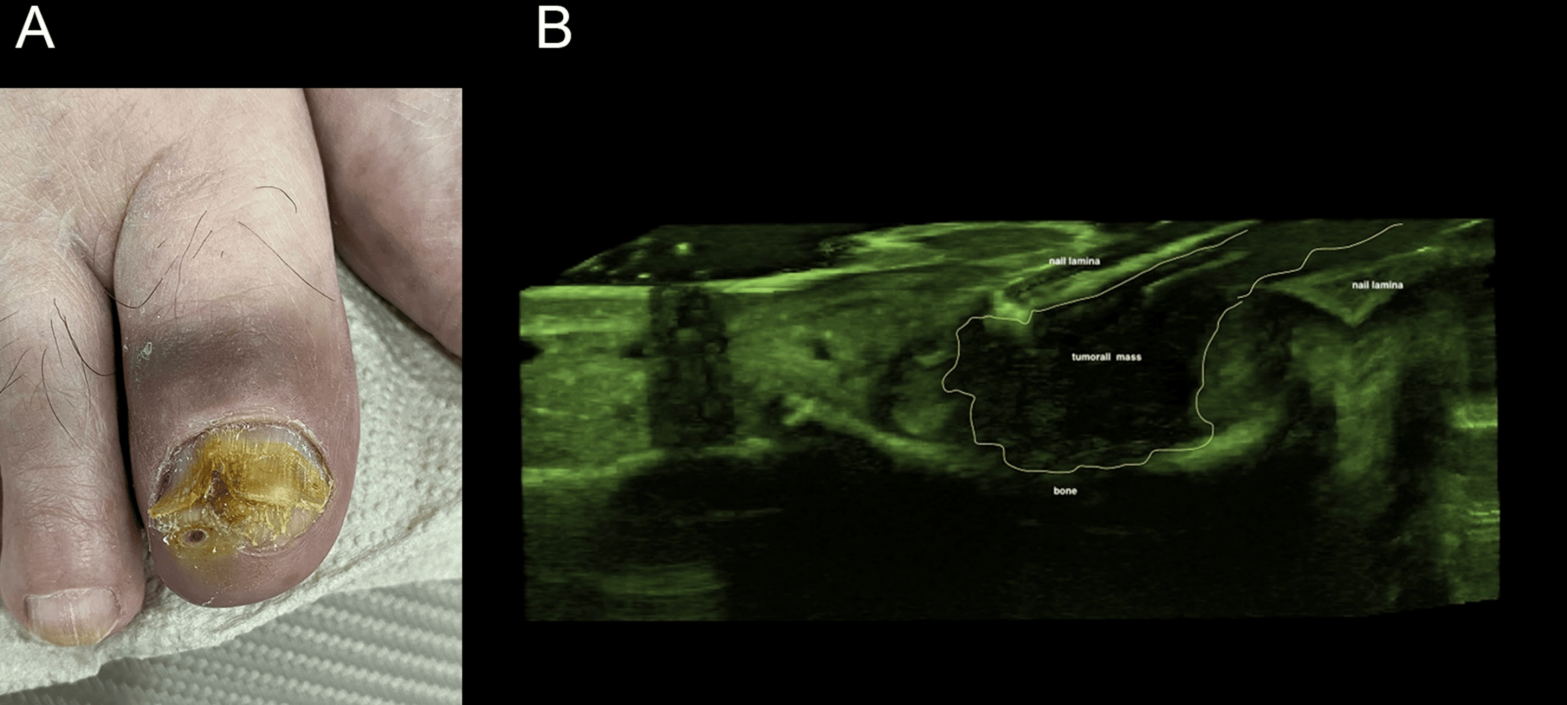
Clinical and ultrasound features of nail squamous cell carcinoma A: shows a clinical image of the right great toenail with the absence of the cuticle, brownish pigmented nail edges, distal onycholysis, a distant fissure in the nail plate, compact subungual hyperkeratosis, and chromonychia; B: 3D HFUS image, delineated in yellow, shows a neoproliferative, hypoechoic, expansive mass causing bone erosion of the distal phalanx and loss of continuity of the nail lamina. Histopathology confirmed squamous cell carcinoma. Original unpublished image, courtesy of Dr. Claudia Gonzalez. HFUS: high-frequency ultrasound

Malignant nail tumors, although rare, pose a significant diagnostic challenge as they frequently mimic benign conditions such as onychomycoses or psoriasis. HFUS plays a crucial role in addressing this challenge by accurately identifying specific ultrasound features indicative of malignancy. These features include distinct vascularization patterns, erosion of the distal phalanx cortex underlying the mass, and changes in the nail plate, as illustrated in Figure 6 [29].

**Figure 7:**
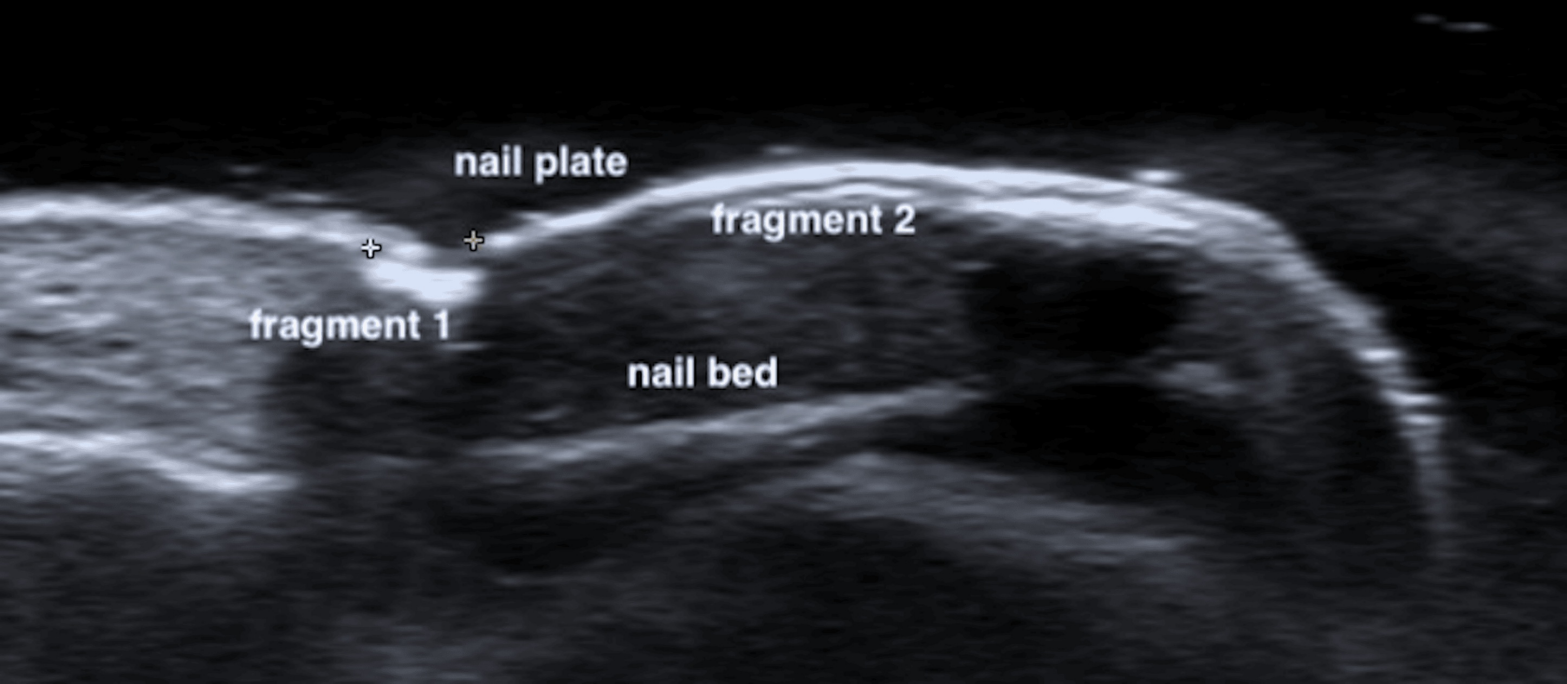
Ultrasound features of onychomadesis Longitudinal greyscale image showing multifragmentary onychomadesis, with two distinct fragments of the nail plate. The first fragment of onychomadesis is delimitated by the + signs. Original unpublished image, courtesy of Dr. Claudia Gonzalez.

HFUS facilitates the evaluation and differentiation of complex mechanical and growth-related NDs. It allows for immediate side-by-side comparison with the unaffected contralateral nail, a technique 72.7% of surveyed clinicians consider essential for accurate diagnosis and management. HFUS enables direct visualization of the nail plate anatomy and its relationship to surrounding soft tissues. This capability confirms diagnoses of conditions such as retronychia, onychomadesis, and onychocryptosis, which might otherwise necessitate a period of observation or surgical intervention, as shown in Figure 7 [30,31].

In summary, HFUS has emerged as a vital adjunct in the evaluation of inflammatory, neoplastic, and nail growth disorders. It is invaluable in distinguishing the nature of a nail lesion (solid vs. cystic), its precise origin (matrix, bed, or periungual tissue), and its extent of invasion, providing clarity in cases where clinical exams and onychoscopy are limited. It effectively reveals subclinical changes, guides clinical management, allows more targeted interventions, and spares patients from unnecessary invasive procedures.

Other radiological imaging for nail disorders

While the primary focus of this study was to assess the knowledge and applications of HFUS in nail pathology, the study also explored the utility of conventional radiography and MRI due to their enduring value in specific scenarios, such as evaluating exostoses and determining the extent of tumors [32,33].

**Figure 8:**
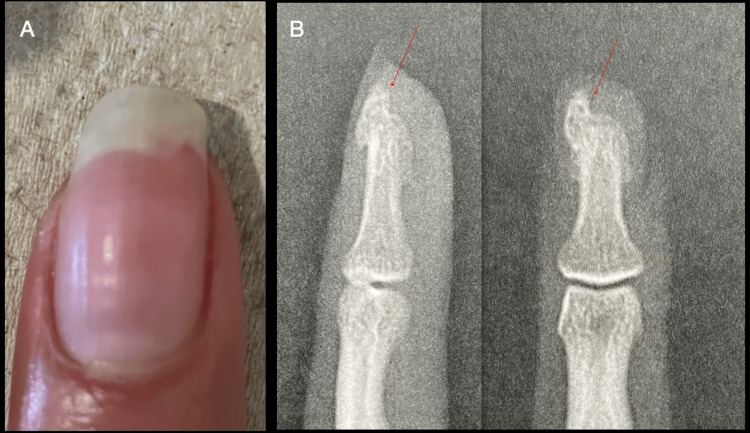
Subungual exostosis A: clinical image of the left index where the patient reported an inability to trim the nail due to its hardness; B: lateral and anteroposterior (AP) projections of the distal phalanx, a red arrow indicates the presence of subungual exostosis. Original unpublished image courtesy of Dr. Claudia Gonzalez.

Subungual exostoses, which are bony origin lesions, are typically identifiable via ultrasound [34,35]. However, for definitive diagnosis, radiography remains the preferred method owing to its ability to clearly depict bone structures. For optimal results, radiographic projections should be performed using a bone technique, ideally magnified, in anteroposterior (AP), lateral, and oblique views. (Figure 8)

There are instances where, if these technical standards are not met, exostoses may not be apparent on the X-ray.

**Figure 9:**
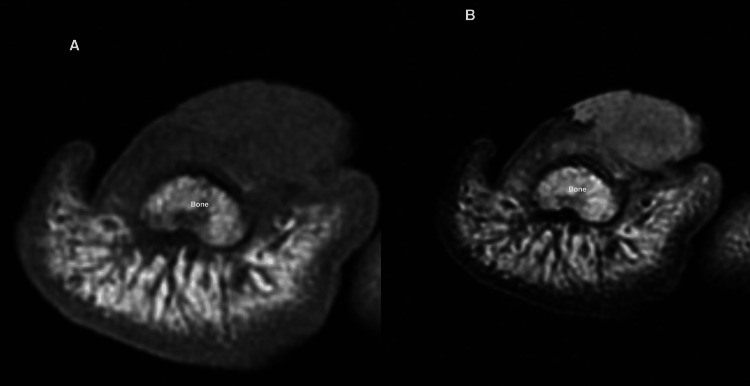
MRI of squamous cell carcinoma of the nail unit A: axial MRI of the nail in a patient diagnosed with squamous cell carcinoma, with the goal to assess the bone extension and the T1-weighted image shows an exophytic mass that is moderately hypointense, involving the entire nail bed and lateral nail fields; B: T1-weighted image with contrast enhancement, which allows for precise delineation of the hyperintense mass. The contrast enhancement confirms that there is no intraosseous extension. Original unpublished image, courtesy of Dr. Claudia Gonzalez.

Regarding the use of MRI in NDs, a significant majority (77.3%) of survey respondents recognized its key applications in determining the intraosseous extension of malignant tumors and ruling out osteomyelitis in cases of NU or related soft tissue infections. These clinical applications are well-supported in the medical literature and highlight the necessity for MRI studies to be conducted with intravenous contrast and using dedicated coils for the hand or foot, as illustrated in Figure 9 [36,37].

Confidence in the ultrasound of the nail unit

The survey results reveal a high level of confidence among healthcare professionals in utilizing HFUS for clinical decision-making. A significant majority, 93%, reported that they always trust the results provided by HFUS when making decisions for their patients. This strong endorsement underscores the reliability and value of HFUS in the diagnostic process of NDs. However, 7% of the respondents expressed a lack of trust in HFUS, suggesting the necessity for further educational initiatives to address potential considerations related to training, familiarity with the technology, or perceived efficacy. This disparity highlights the need not only to continue promoting the benefits and applications of HFUS but also to address the concerns of those who remain skeptical. Targeted training programs to cover both the technical aspects and clinical applications of HFUS more comprehensively could help increase confidence levels across all users. Such initiatives would ensure that HFUS is not only widely adopted but also effectively utilized in clinical practice, maximizing its potential benefits in diagnosing and managing NDs.

The cross-sectional design of this study has inherent limitations, including the possibility of selection bias and the inability to assess changes in practice over time. Additionally, while the response rate achieved provides a substantial basis for analysis, a larger sample size could have enhanced the generalizability of the findings and provided a more comprehensive understanding of HFUS applications across a wider range of clinicians. Nevertheless, the responses gathered are representative of a diverse group of specialists and offer significant insights into the current clinical practices and perspectives regarding the use of HFUS in ND diagnosis. Future research could benefit from a longitudinal design to better understand the evolution of clinical practices and the integration of HFUS over time. Increasing the sample size and encouraging a higher response rate would also strengthen the statistical power of future studies of HFUS use in both routine and complex ND evaluations.

## Conclusions

HFUS has proven to be an indispensable diagnostic tool in the evaluation of nail pathologies, offering unparalleled accuracy and detailed insights into both inflammatory and neoplastic conditions. This study provides a fundamental understanding of the current use and perceived value of HFUS in the diagnosis and management of NDs. The findings highlight the importance of HFUS as a non-invasive, high-resolution imaging modality and identify key areas for future educational initiatives to enhance clinicians’ skills and confidence in using this valuable tool. As HFUS continues to be integrated into clinical practice, it promises to significantly advance the standard of care in dermatology by enabling more targeted and effective treatment strategies for patients suffering from NDs.

## Appendices

Appendix 1 

**Table 2:** Ultrasound evaluation of the nail unit survey High-frequency ultrasound (HFUS) is increasingly recognized as a valuable tool in the diagnosis and treatment of nail disorders. This survey aims to assess current HFUS clinical practices and identify opportunities to enhance interpretation and diagnostic accuracy in this area. Please select the response you consider correct.

Question	Choices
1. What is the imaging method of choice to assess nail diseases?	a. Magnetic resonance imaging (MRI)
	b. High frequency ultrasound (HFUS)
	c. X-rays
	d. Computed tomography (CT)
	e. Diagnostic imaging is not useful in nail diseases.
2. What type of transducer should be used to perform nail unit ultrasound?	a. Linear 7 MHz
	b. Linear 12 MHz
	c. Convex 12 MHz
	d. Linear 15 MHz
	e. Linear 70 MHz
3. High-resolution nail ultrasound should be performed:	a. In grayscale only
	b. Using Doppler, duplex, color
	c. In grayscale and using Doppler/duplex color
	d. It is not useful to perform nail ultrasound
4. For which conditions is comparative nail ultrasound always recommended?	a. Retronychia
	b. Psoriasis
	c. Lichen planus
	d. Comparative nail ultrasound should always be performed
	e. Comparative nail ultrasound should never be performed
5. What are the requirements for a proper nail ultrasound assessment?	a. Trained physician in nail disorders
	b. High-resolution ultrasound with Doppler analysis
	c. Copious amount of gel
	d. Study in grayscale and color Doppler
	e. All the above
6. How many assessments per year should a physician complete to demonstrate competence in performing nail ultrasound?	a. 10 studies per year
	b. 30 studies per year
	c. 100 studies per year
	d. 300 studies per year
	e. None of the above
7. In which of the following disorders is nail ultrasound indicated?	a. Psoriasis
	b. Lichen planus
	c. Viral wart
	d. All of the above
	e. None of the above
8. In which of the following pathologies is nail ultrasound useful?	a. Benign tumors
	b. Malignant tumors
	c. Subungual exostoses
	d. Myxoid cysts
	e. All of the above
	f. None of the above
9. In which of the following disorders is nail ultrasound of utility?	a. Retronychia
	b. Onychomadesis
	c. Onychocryptosis
	d. All of the above
	e. None of the above
10. How should an X-ray be requested to study exostoses?	a. Comparative X-ray of the feet
	b. X-ray of the affected nail unit, anteroposterior (AP) and lateral views
	c. X-ray of the affected nail unit, AP, lateral, and oblique views
	d. Magnified X-ray of the affected nail unit, AP, lateral, and magnified oblique views
11. How should an MRI be requested to study nail diseases?	a. Simple comparative MRI of the feet
	b. Contrast-enhanced comparative MRI of the feet
	c. Non-contrast-enhanced MRI with a dedicated foot coil of the affected nail
	d. Contrast-enhanced MRI with a dedicated foot coil of the affected nail
12. In what nail disorders is MRI of the nail unit indicated?	a. Suspected medullary involvement by cancer
	b. Invasive melanoma
	c. Osteomyelitis
	d. All of the above *
	e. None of the above
13. How often do you request a nail unit ultrasound?	a. Frequently
	b. Rarely
	c. Whenever there is an indication
	d. I do not find ultrasound useful for nail pathology
14. How much do you trust the results provided by a high-resolution ultrasound to make decisions for your patient?	a. Always trust
	b. Never trust
15. What is your medical specialty?	a. Dermatologist
	b. Radiologist
	c. Pathologist
	d. Rheumatologist
